# Supplementary material for: Computational 3D histological phenotyping of whole zebrafish by X-ray histotomography
Source: eLife. 2019 May 7;8:e44898. doi: 10.7554/eLife.44898 (PMC6559789; doi:10.7554/eLife.44898)
Supplement: Transparent reporting form [file elife-44898-transrepform.docx]

***eLife’s* transparent reporting form**

We encourage authors to provide detailed information *within their submission* to facilitate the interpretation and replication of experiments. Authors can upload supporting documentation to indicate the use of appropriate reporting guidelines for health-related research (see [EQUATOR Network](http://www.equator-network.org/%20)), life science research (see the [BioSharing Information Resource](https://biosharing.org/)), or the [ARRIVE guidelines](http://www.plosbiology.org/article/info:doi/10.1371/journal.pbio.1000412) for reporting work involving animal research. Where applicable, authors should refer to any relevant reporting standards documents in this form.

If you have any questions, please consult our Journal Policies and/or contact us: [editorial@elifesciences.org](mailto:editorial@elifesciences.org).

**Sample-size estimation**

- You should state whether an appropriate sample size was computed when the study was being designed
- You should state the statistical method of sample size computation and any required assumptions
- If no explicit power analysis was used, you should describe how you decided what sample (replicate) size (number) to use

Please outline where this information can be found within the submission (e.g., sections or figure legends), or explain why this information doesn’t apply to your submission:

The primary goal of the study was to demonstrate the feasibility of synchrotron micro-CT for high-throughput 3D histology, thus the exact sample size needed was not computed at the time the study was designed. We aimed to test and optimize a variety of samples at the synchrotron. Additionally, since beamline time is a competitive application process, sample sizes were kept small. A sample size of 5 for the brain-specific nuclear count experiment was determined by the availability of scanned and reconstructed samples at the time of analysis.

**Replicates**

- You should report how often each experiment was performed
- You should include a definition of biological versus technical replication
- The data obtained should be provided and sufficient information should be provided to indicate the number of independent biological and/or technical replicates
- If you encountered any outliers, you should describe how these were handled
- Criteria for exclusion/inclusion of data should be clearly stated
- High-throughput sequence data should be uploaded before submission, with a private link for reviewers provided (these are available from both GEO and ArrayExpress)

Please outline where this information can be found within the submission (e.g., sections or figure legends), or explain why this information doesn’t apply to your submission:

Qualitative analysis of *hht* mutants versus wild-type were done for all acquired *hht* zebrafish (1x at 2,3,4-dpf, and 2x at 5-dpf). 5 wild-type zebrafish samples from the same clutch of eggs were used for counting brain nuclei as mentioned in the text (See Methods). Nuclear counts for each brain region were computed once and directly reported along with volumes in Tables 1 and 2.

**Statistical reporting**

- Statistical analysis methods should be described and justified
- Raw data should be presented in figures whenever informative to do so (typically when N per group is less than 10)
- For each experiment, you should identify the statistical tests used, exact values of N, definitions of center, methods of multiple test correction, and dispersion and precision measures (e.g., mean, median, SD, SEM, confidence intervals; and, for the major substantive results, a measure of effect size (e.g., Pearson's r, Cohen's d)
- Report exact p-values wherever possible alongside the summary statistics and 95% confidence intervals. These should be reported for all key questions and not only when the p-value is less than 0.05.

Please outline where this information can be found within the submission (e.g., sections or figure legends), or explain why this information doesn’t apply to your submission:

The brain nuclei count for 5x wild-type 5 dpf zebrafish was reported. The average (arithmetic mean) and standard deviation of these five brain nuclei counts along with their raw values were reported in Table 2. Table 3 reports the volumetric sizes of the 5x 5 dpf zebrafish along with their arithmetic mean and standard deviation.

(For large datasets, or papers with a very large number of statistical tests, you may upload a single table file with tests, Ns, etc., with reference to sections in the manuscript.)

**Group allocation**

- Indicate how samples were allocated into experimental groups (in the case of clinical studies, please specify allocation to treatment method); if randomization was used, please also state if restricted randomization was applied
- Indicate if masking was used during group allocation, data collection and/or data analysis

Please outline where this information can be found within the submission (e.g., sections or figure legends), or explain why this information doesn’t apply to your submission:

As stated in the Methods, mutant *hht* zebrafish were screened around day 3 and

No masking or randomization was used for group allocation, data collection, or analysis.

**Additional data files (“source data”)**

- We encourage you to upload relevant additional data files, such as numerical data that are represented as a graph in a figure, or as a summary table
- Where provided, these should be in the most useful format, and they can be uploaded as “Source data” files linked to a main figure or table
- Include model definition files including the full list of parameters used
- Include code used for data analysis (e.g., R, MatLab)
- Avoid stating that data files are “available upon request”

Please indicate the figures or tables for which source data files have been provided:

Tables 2 and 3 provide the raw numerical data used in Figures 8 and 9. The supporting data for those tables (including raw counts, nucleus position) is found in the Dryad Digital Repository (<https://doi.org/10.5061/dryad.4nb12g2>).

Since each full resolution data set can be around 100 GB, we have shared image files using, ViewTool, which is publicly available at <http://3D.fish>. Full data sets in their original format are available by request. The available files are shown in Table 1. Registered and unregistered 8-bit reconstructions of the heads of five zebrafish larvae involved in analysis are also available in the Dryad repository linked above, as well as scripts written for cell nuclei detection, analysis, and sample registration.
